# Low-Dose Decitabine Monotherapy Reverses Mixed Chimerism in Adult Patients After Allogeneic Hematopoietic Stem Cell Transplantation With Myeloablative Conditioning Regimen: A Pilot Phase II Study

**DOI:** 10.3389/fmed.2021.627946

**Published:** 2021-02-23

**Authors:** Ling Wang, Li-ning Wang, Ji-fang Zhou, Wen-hui Gao, Chuan-he Jiang, Wei Tang, Wei-li Zhao, Jiong Hu, Jie-ling Jiang

**Affiliations:** ^1^Department of Hematology, Blood and Marrow Transplantation Center, Collaborative Innovation Center of Hematology, Shanghai Institute of Hematology, Rui Jin Hospital, Shanghai Jiao Tong University School of Medicine, Shanghai, China; ^2^School of International Pharmaceutical Business, China Pharmaceutical University, Nanjing, China

**Keywords:** allogeneic stem cell transplantation, low-dose decitabine, mixed chimerism, pre-emptive therapy, myeloablative conditioning regimen

## Abstract

T cell mixed chimerism (MC) after allogeneic hematopoietic stem cell transplantation (allo-HSCT) with myeloablative conditioning for hematological malignancies may indicate engraftment failure or disease relapse. Immune modulation, such as donor lymphocyte infusion (DLI) or the rapid tapering-off or stopping of immunosuppressive treatment, can reverse MC to full donor chimerism (FDC). However, the development or aggravation of graft-versus-host disease (GvHD) and the related mortality remain major concerns with immune modulation. In this prospective, single-arm study (NCT03663751), we tested the efficacy and safety of low-dose decitabine (LD-DAC, 5 mg/m^2^ daily for 5 days and repeated every 6–8 weeks) without immune modulation in the treatment of patients with MC to prevent MC-associated relapse and/or graft failure. A total of 14 patients were enrolled. All the patients received myeloablative conditioning regimens, and MC was documented from day +30 to day +180 after allo-HSCT with a donor chimerism level ranging from 59 to 97% without detectable measurable residual disease (MRD). Eleven patients (78.6%) responded favorably to treatment, showing increased levels of donor chimerism (≥95%), while nine achieved FDC. All of these patients maintained their responses for a median of 11 months (3–22). The three patients who failed to respond favorably eventually either relapsed or experienced graft failure. All three were alive and in remission at the last follow-up after the second allo-HSCT. LD-DAC monotherapy was well tolerated and exerted limited hematological and nonhematological toxicities. New-onset GvHD symptoms were observed only in two patients. Overall, the estimated 2-year overall survival (OS) and event-free survival (EFS) after allo-HSCT were 90.9 ± 8.7% and 67.0 ± 13.7%, respectively. In conclusion, LD-DAC alone could reverse MC in most patients after allo-HSCT with myeloablative conditioning, while those who achieved FDC enjoyed long-term EFS without major complications. Further prospective studies with larger sample sizes are warranted to confirm the benefits of LD-DAC.

## Introduction

Allogeneic stem cell transplantation (allo-HSCT) is a potentially curative therapy for malignant hematological diseases. Disease relapse remains a major cause of treatment failure (1, 2). The monitoring of disease-related parameters, such as measurable residual disease (MRD), can detect evidence of low-volume disease, which can serve as an indicator for emerging relapse (3). Hematopoietic chimerism analysis, which can distinguish residual recipient hematopoiesis from donor cells, is useful for the monitoring of allograft health and predicting imminent graft rejection, and can also be an indicator of potential relapse (2). The gold standard for quantitative chimerism analysis relies on the polymerase chain reaction (PCR)-based detection of variable number tandem repeats (VNTRs) or short tandem repeat (STR) polymorphisms in DNA from bone marrow or peripheral blood mononucleated cells or T cells, as recommended by the EuroChimerism Consortium (4–7).

Several studies have demonstrated that patients with mixed chimerism (MC) in either mononucleated cells or CD3+ T cells display a significantly higher incidence of relapse (40–90%) than those with complete donor chimerism (10–20%). The time between the detection of MC and relapse (median ~70 days) may permit timely therapeutic intervention (8–10). The rapid withdrawal of immunosuppression (RWIS) and preemptive donor lymphocyte infusion (DLI) may result in full donor chimerism (FDC) and are effective in reducing the relapse rate (RR) (11–14). However, RWIS and DLI have also been associated with complications, such as the development or aggravation of graft-versus-host disease (GvHD). Notably, MC without MRD does not necessarily equate to disease recurrence because the recrudescence of host hematopoiesis may represent normal hematopoiesis. In such a scenario, clinical decisions of immune modulation are complicated owing to the unnecessary risk of aggravation of GvHD (1, 2).

Epigenetic modulation of histone deacetylases (HDCs) such as sirturin-1 or methylation is important in maintaining normal function of hematopoietic stem cells and potentially regulating GvHD or graft versus leukemia effect (GvL) in the allo-HSCT settings (15–17). Hypomethylating agents (HMAs), administered either prophylactically or preemptively, are important treatment options after allo-HSCT for patients with acute myeloid leukemia (AML) or myelodysplasia (MDS) (18, 19). Multiple studies have demonstrated that HMAs exert significant immunomodulatory effects and are important for reducing post-transplantation relapse, and do so without inducing GvHD (20). In our previous study, we observed that low-dose decitabine (LD-DAC) converted MC into FDC in patients during maintenance therapy (21). In this pilot prospective study, we assessed the efficacy and safety of LD-DAC in the treatment of patients with MC in CD3+ T cells after allo-HSCT with myeloablative conditioning (MAC).

## Methods

### Study Design

This was an investigator-initiated, prospective, nonrandomized, single-arm, phase II clinical trial (NCT 03663751) to evaluate the efficacy and safety of LD-DAC as a monotherapy for patients with MC and who were also MRD-negative after allo-HSCT. The study was approved by the Human Ethics Committee of the Rui Jin Hospital and was conducted in accordance with the Declaration of Helsinki. The study was conducted in the Blood and Marrow Transplantation Center, Department of Hematology, Rui Jin Hospital, Shanghai Jiao Tong University School of Medicine. All enrolled patients provided written informed consent.

### Study Protocol

The inclusion criteria were as follows: (1) adult patients (16–60) undergoing allo-HSCT with myeloablative conditioning from human leukocyte antigen (HLA)-matched sibling donors (MSDs), matched unrelated donors (MUDs), or haploidentical (Haplo) donors; (2) patients who achieved hematological engraftment and presented with a sustainable absolute neutrophil count (ANC) of >0.5 × 10^9^/L not dependent on granulocyte colony-stimulating factor; (3) patients with hematological malignancies and presenting with measurable disease as indicated by immunophenotyping and/or molecular analysis; and (4) patients presented with MC (<99%) among T cells from either bone marrow or peripheral blood and who were MRD-negative in the bone marrow (<0.01%) after transplantation. The exclusion criteria were (1) patients with grade II-IV acute GvHD (aGvHD) or moderate to severe chronic GVHD (cGvHD) not responding to the treatment and (2) patients with severe complications such as life-threatening infections (bacterial, viral, or fungal), sinusoid obstructive syndrome (SOS), HSCT-associated thrombotic microangiopathy (TA-TMA), or posterior reversible encephalopathy syndrome (PRES) not responding to treatment.

**Figure 1:**
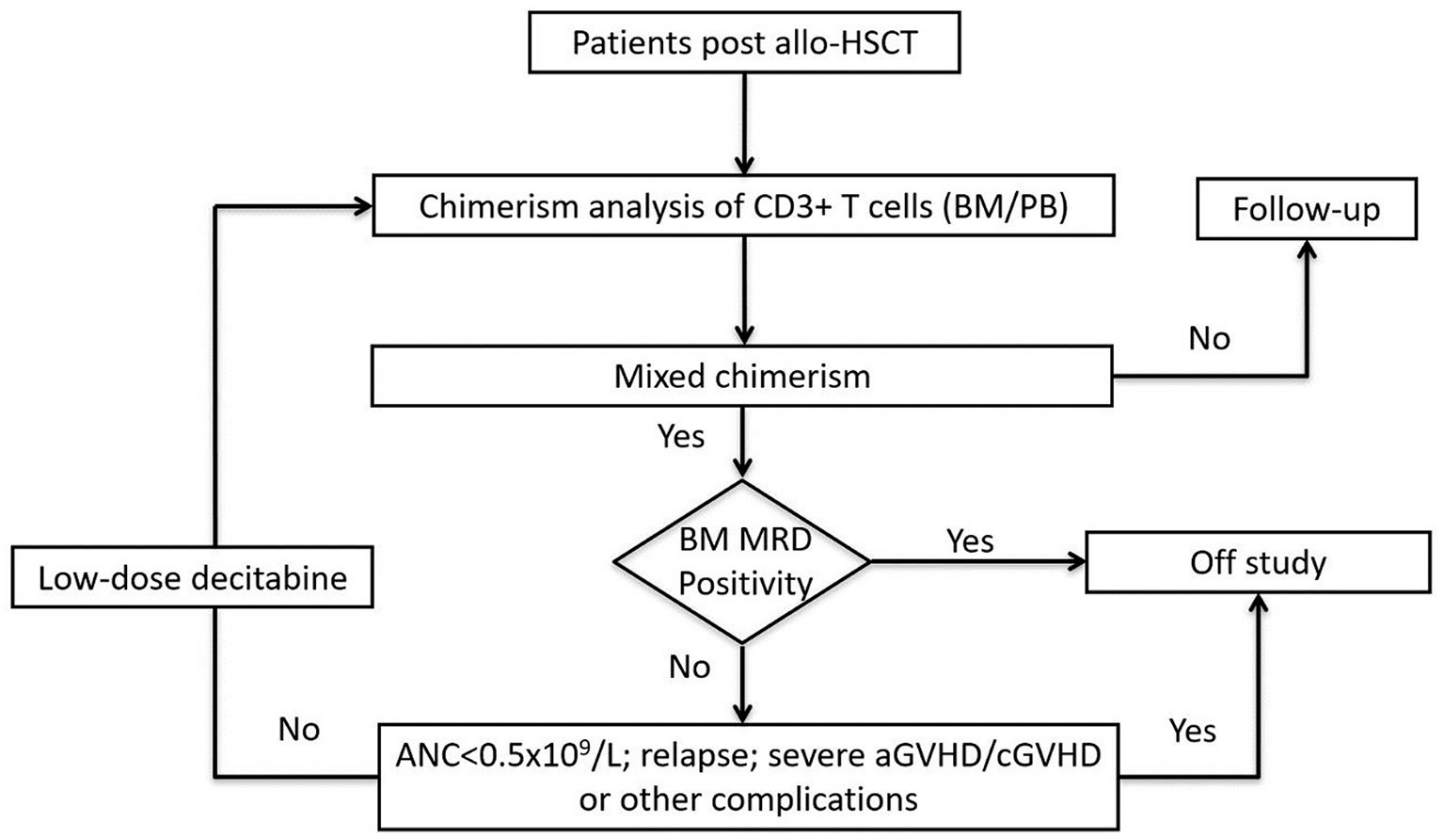
Flow chart of the study design.

All the enrolled patients received LD-DAC at 5 mg/m^2^ for 5 consecutive days. Chimerism monitoring was performed 4 weeks after the treatment. Patients who showed a favorable response were followed-up every 6–8 weeks. For patients who did not achieve a favorable response, the LD-DAC treatment was repeated every 6–8 weeks for up to 4 cycles. For patients undergoing immunosuppressive (IS) treatment, either as prophylaxis or for ongoing GvHD, RWIS was not implemented. For patients not receiving immunosuppressive treatment, no immunomodulatory therapy, such as interferon administration or DLI, were allowed. Patients were removed from the trial in the case of events such as the emergence of MRD, relapse, new onset, or aggravation of existing GvHD to grade III–IV aGvHD or moderate/severe cGvHD, life-threatening infection, SOS, TA-TMA, PRES, or other severe HSCT-associated complication (Figure 1).

### Definition of Response and Study Endpoints

Responses were assigned as follows: (1) Complete response (CR): patients achieving FDC (≥99%); (2) major response (MR): patients with increased donor chimerism (≥95%); (3) partial response (PR): patients with a 10% increase in the level of donor chimerism but that failed to reach 95%; and (4) no response (NR): patients with no or less than a 10% increase in the level of donor chimerism, and that failed to reach 95%. CRs and MRs were considered favorable, whereas PRs or NRs were considered unfavorable.

The primary endpoint of the study was achieving favorable responses (CR and MR) at 6 months after enrollment. Secondary endpoints included unfavorable events, namely, newly developed grade III–IV aGvHD or moderate to severe cGvHD, graft failure, relapse, or nonrelapse mortality (NRM) documented 6 months after enrollment; and survival data, including overall survival (OS), RR, NRM, and event-free survival (EFS) at 2 years after allo-HSCT.

### Chimerism Analysis

Genomic DNA was extracted from 200 μL of CD3+ T cells (EDTA-treated) obtained from whole blood or bone marrow. A total of 25 ng of the extracted DNA was used for the amplification of 16 autosomal STRs (D8S1179, D21S11, D7S820, CSF1PO, D3S1358, D5S818, D13S317, D16S539, D2S1338, D19S433, VWA, D12S391, D18S51, Amel, D6S1043, and FGA) using the AmpFLSTR® Huaxia™ PCR Direct Amplification Kit (Invitrogen, Beijing, China). A total of 0.5 μL of the amplified product was mixed with 9 μL of Hi-Di formamide and 0.5 μL of GeneScan-500 Liz molecular weight marker for electrophoresis run on an ABI 3130 Genetic Analyzer (Applied Biosystems, Foster City, CA, USA). GeneMapper1 v3.2.1 was adopted to analyze the genotype of each site based on the length of the DNA fragments and allelic ladders. The chimerism values were calculated from the observed peak areas of the informative markers. The calculation procedure was standardized to obtain reproducible chimerism values. The length of the labeled recipient and donor alleles was determined through the analysis of donor and recipient DNA isolated before transplantation. The allele lengths of all the markers were scored. The relative positions of the donor and recipient alleles of a given marker determined its applicability to the calculation of mosaicism, as described by Nollet et al. (22). Chimerism analysis had a sensitivity of 1% and ≥99% was considered to be FDC.

### Sample Size Estimation and Statistical Analysis

This was a phase II study based on Simon's two-stage design (23). The study hypothesis was based on an expected favorable response rate ≥80% with an unacceptable favorable response rate ≤50%. The trial would be stopped early if the number of patients showing a favorable response failed to meet the relevant criteria (Supplementary Table 1).

Concerning safety, severe unfavorable events were defined as detectable MRD or disease relapse, graft failure, NRM of any cause, newly developed or aggravated aGvHD to grade III–IV or moderate to severe cGvHD, life-threatening infections, or other allo-HSCT-associated complications such as SOS, TA-TMA, and PRES. A 30% value was set as the unacceptable level of overall incidence of severe adverse events following, which the study would be stopped early based on the continuous monitoring for toxicity using Pocock-type boundary (Supplementary Table 2) (24). Survival rates were calculated using Kaplan–Meier estimates (25). OS was calculated from day 0 to the date of death from any cause. EFS was calculated from day 0 to the date of occurrence of aGVHD (III–IV) or moderate to severe cGVHD, graft failure, relapse, or death of all causes.

## Results

### Patient Characteristics

A total of 14 patients were enrolled in the study. All the patients had hematological malignancies and received MAC mostly with fludarabine (150 mg/m^2^) and busulfan (12.8 mg/kg) or sequential high-dose chemotherapy (cladribine + cytarabine + etoposide) followed by fludarabine (150 mg/m^2^) and busulfan (9.6 mg/kg) conditioning. For myeloid leukemia, GvHD prophylaxis was a standard regimen comprising cyclosporin plus methotrexate and mycophenolate mofetil, with anti-thymoglobulin (ATG) 6 or 10 mg/kg for HLA-MUD or mismatched related donor transplantation. For patients with lymphoid malignancies, GvHD prophylaxis was post-transplantation cyclophosphamide [50 mg/(kg·day^−1^) at days +3 and +4] with tacrolimus starting from day+5 or low-dose ATG [2.5 mg/(kg·day^−1^) at day +15 or after neutrophil engraftment in MUD and haplo settings]. All these patients achieved negative MRD day 28–30 after allo-HSCT and remained negative when enrolled in this study. The characteristics of the patients are shown in Table 1.

**Table 1:** Patient characteristics and outcomes.

**UPN**	**#1**	**#2**	**#3**	**#4**	**#5**	**#6**	**#7**	**#8**	**#9**	**#10**	**#11**	**#12**	**#13**	**#14**
Age	45	20	45	51	20	51	16	40	29	20	60	20	20	16
Sex	M	M	F	F	M	F	M	M	M	M	M	F	M	M
Diagnosis	CML	ALL	AML	MDS-EB2	ALL	AML	ALL	AML	Sezary	T-NHL	AML	AML	Ph+ ALL	ALL
Disease status at transplant	CP3- T315I	CR1	CR1	NR	CR1	CR1	CR1	CR2	NR	CR3	MRD+ CR1	MRD+ CR1	CR1	CR1
Donor type	Haplo	MUD	Sib	MUD	Sib	Sib	Haplo	Sib	MUD	Haplo	Sib	MUD	Haplo	Sib
GvHD status	Skin cGvHD	/	/	/	/	/	/	/	/	/	/	/	/	/
IS prophylaxis	FK506	FK506	/	CsA	FK506	/	/	/	/	FK506	FK506	FK506	FK506	FK506
Time of MC	+90	+60	+100	+60	+76	+120	+101	+157	+180	+92	+35	+75	+30	+30
MRD level	–	–	–	–	–	–	–	–	–	–	–	–	–	–
DC level	89%	97%	82%	76%	89%	93%	91%	89%	93%	92%	81%	91%	59%	85%
DC after the first LD-DAC	>99%	95%	89%	94%	81%	>99%	95%	>99%	96%	95%	93%	97%	2%	98%
DC after the second LD-DAC	/	>99%	>99%	86%	90%	/	95%	/	>99%	>99%	>99%	99%	/	/
DC after the third LD-DAC	/	/	/	86%	96%	/	2%	/	/	/	/	/	/	/
DC level at six-month	>99%	>99%	>99%	86%	96%	>99%	2%	>99%	>99%	>99%	>99%	99%	0	98%
Six-month response	CR	CR	CR	PR	MR	CR	NR	CR	CR	CR	CR	CR	NR	MR
GvHD^*^	–	–	–	+	–	–	–	–	–	–	+	–	–	–
Relapse^**^	–	–	–	+	–	–	–	–	–	–	–	–	–	–
Graft failure^***^	–	–	–	–	–	–	+	–	–	–	–	–	+	–
NRM	–	–	–	–	–	–	–	–	–	–	+	–	–	–
Inremission	+	+	+	+	+	+	+	+	+	+	–	+	+	+
OS (days)	878+	822+	803+	709+	546+	529+	509+	456+	418+	287+	372	321+	257+	130+

*UPN, unique patient number; CML, chronic myelocytic leukemia; ALL, acute lymphoblastic leukemia; AML, acute myeloid leukemia; MDS EB2, myelodysplastic syndrome with excess blasts 2; T-NHL, T cell non-Hodgkin's lymphoma; Ph+, Philadelphia chromosome-positive; CP3, third chronic phase; CR1/2/3, first/second/third complete remission; NR, nonremission; MRD+, measurable residual disease-positive; Haplo, haploidentical; MUD, matched unrelated donor; Sib, sibling; cGvHD, chronic graft versus host disease; IS, immunosuppressor; CsA, cyclosporine A; MC, mixed chimerism; DC, donor chimerism; LD-DAC, low-dose decitabine; CR, complete response; PR, partial response; MR, major response; NR, no response; NRM, nonrelapse mortality; OS, overall survival*.

* *UPN#4 presented newly developed grade II acute GvHD (skin rash) which progressed to chronic skin GvHD; UPN#11 developed bronchiolitis obliterans (BO) 4 months after the second cycle of LD-DAC and eventually died of a lung infection*.

** *Relapse: UPN#4 progressed to AML on day +335 and received azacytidine plus venetoclax followed by a second allogeneic hematopoietic stem cell transplantation (allo-HSCT)*.

*** *UPN#7 and UPN#13 both developed graft failure after ganciclovir treatment of CMV DNAemia. UPN#7 subsequently received a second allo-HSCT from a MUD and was in remission 9 months later; UPN#13 underwent a second allo-HSCT from a MUD and was in remission 2months after the second allo-HSCT*.

MC was documented from day +30 to day +180 after allo-HSCT, and the donor chimerism level ranged from 59 to 97%. A total of 26 cycles of LD-DAC were given. Five patients received one cycle of LD-DAC, six patients received two, and three received three.

### Response to LD-DAC Treatment

**Figure 2:**
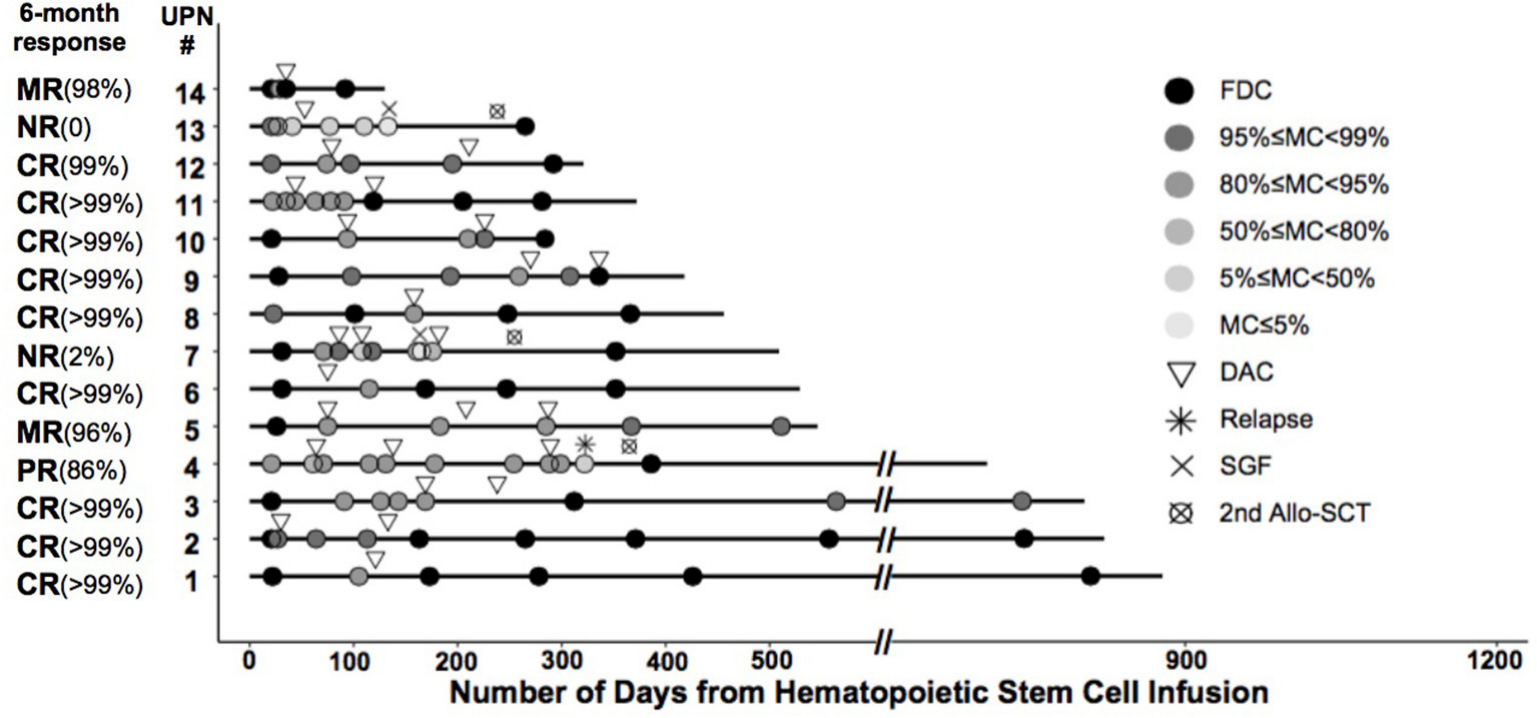
Timing and outcome of chimerism analysis among the enrolled patients. Shown is the chimerism outcome for each patient enrolled in the study at different time points. The level of donor chimerism is indicated by the color of the closed circle. The inverted triangle indicates the time of low-dose decitabine (LD-DAC) treatment given for each patient.

A total of 11 patients showed a favorable response (78.6%). Nine of them achieved CR (FDC ≥ 99%)—three after one cycle of LD-DAC, and six after two cycles. Two patients achieved MR (>95% of donor chimerism) after one and three cycles of LD-DAC, respectively. Notably, at 6 months after enrollment or the last follow-up, all of these patients maintained their responses for a median of 372 days (132–780; Figure 2).

Three patients failed to reach a sustainable favorable response. Patient #4 achieved only a PR after two cycles of LD-DAC, with a donor chimerism level of 94%. Patient #7 had a quick MR after the first cycle of LD-DAC but rapidly experienced graft failure after ganciclovir treatment for cytomegalovirus (CMV) reactivation. Patient #13, with 59% donor chimerism, failed to respond and had graft failure 3 weeks after one cycle of LD-DAC (Figure 2).

Overall, 6 months after enrollment, 10 patients maintained a favorable response without major unfavorable events. Four patients had unfavorable events, including relapse with initial PR (patient #4), loss of initial response with graft failure (patient #7), NR with graft failure (patient #13), and development of moderate to severe cGvHD and NRM (patient #11).

### Toxicity and Complications

Each adverse event was graded according to the National Cancer Institute's Common Terminology Criteria for Adverse Events (NCI-CTCAE) version 5.0. For the 26 cycles of LD-DAC given, including the two that resulted in graft failure, grade III–IV hematological toxicities were observed, including neutropenia and thrombocytopenia (Table 2). If the two cycles that resulted in graft failure are excluded, the grade IV incidence of neutropenia and thrombocytopenia was 33.3% for both parameters. All the patients recovered rapidly, mostly within 7 days, and no life-threatening neutropenic fever and/or bleeding episodes were observed. No severe nonhematological toxicities were documented.

**Table 2:** Hematological toxicities after LD-DAC treatment (26 cycles).

	**WBC (%)**	**ANC (%)**	**Hb (%)**	**PLT (%)**
None	0	2 (7.7)	1 (3.8)	1 (3.8)
Grade 1	3 (11.5)	3 (11.5)	8 (30.8)	4 (15.4)
Grade 2	7 (26.9)	5 (19.2)	7 (26.9)	4 (15.4)
Grade 3	6 (23.1)	7 (26.9)	9 (34.6)	8 (30.8)
Grade 4	10 (38.5^*^)	9 (34.6^*^)	1 (3.8^*^)	9 (34.6^*^)

*LD-DAC, low-dose decitabine; WBC, white blood cell count; ANC, absolute neutrophil count; Hb, hemoglobin; PLT, platelet count*.

* *If the two cycles of treatment that were associated with graft failure are excluded, the grade IV toxicities were 9 (37.5%), 8 (33.3%), 0, and 8 (33.3%) for WBC, ANC, Hb, and PLT, respectively*.

There was no aggravation of existing GvHD (patient #1). Patient #4 experienced a new-onset skin rash 4 days after the first LD-DAC treatment, which developed into cGVHD that did not respond well to tacrolimus and was eventually controlled with sirolimus. Patient #11 developed symptoms of dyspnea 4 months after the second LD-DAC treatment and was later diagnosed with bronchiolitis obliterans (BO). Otherwise, there were no life-threatening infections, SOS, TA-TMA, PRES, or other severe complications associated with the HSCT.

### Follow-Up Outcome

At the last follow-up on September 30, 2020, the median time of follow-up was 526 days after allo-HSCT (130–878) and 372 days (132–780) after enrollment. A total of 13 patients were alive, including 10 without disease relapse or progression.

**Figure 3:**
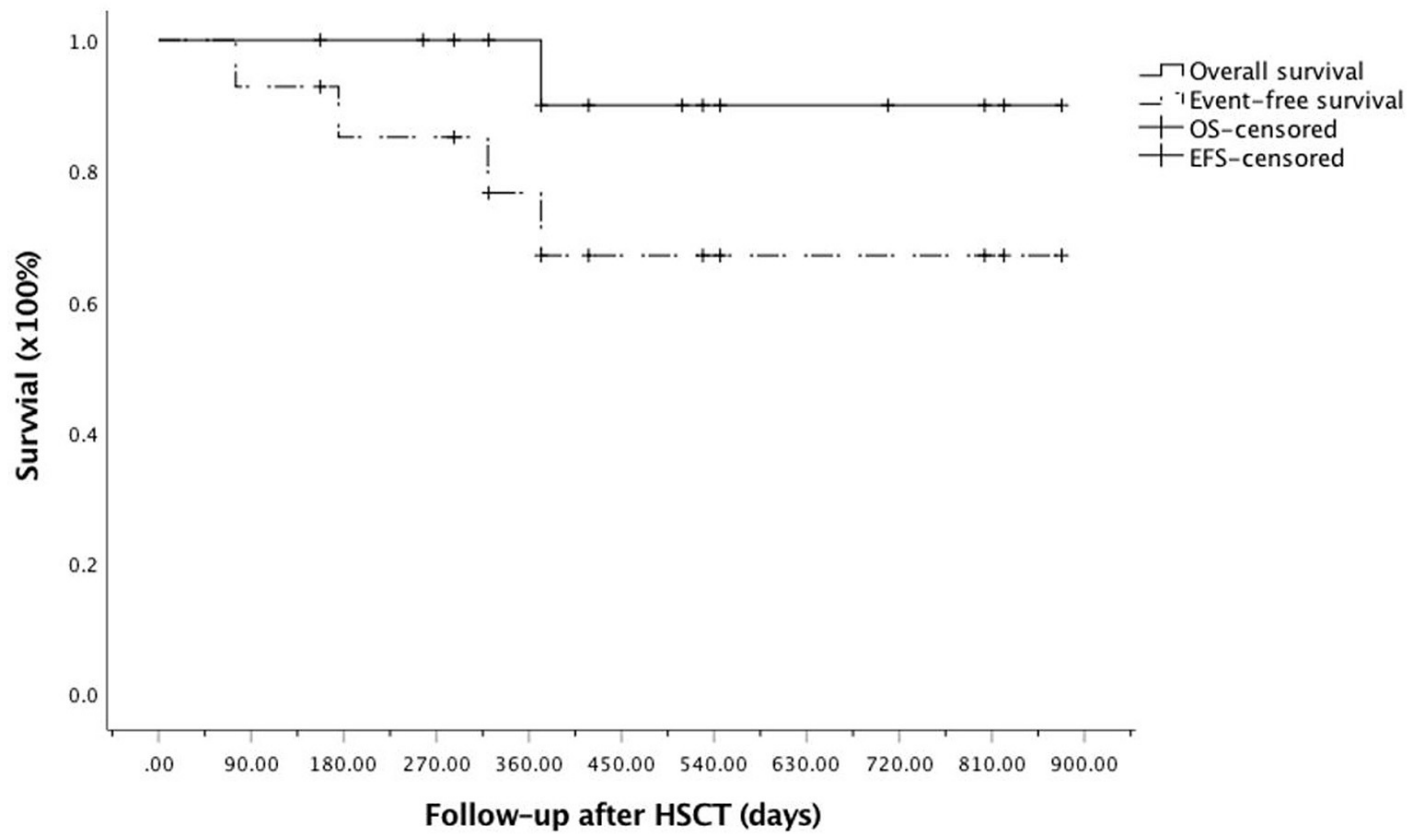
Kaplan–Meier curve for overall survival (OS) and event-free survival (EFS) after allogeneic hematopoietic stem cell transplantation (allo-HSCT) among the enrolled patients. OS, solid line; EFS, dotted line.

Three patients were removed from the study owing to relapse and/or secondary graft failure. Patient #4, who had MDS, progressed to AML 11 months after the first MUD allo-HSCT. The patient was rescued by azacytidine plus venetoclax treatment followed by a second Haplo donor allo-HSCT and was alive and in remission 12 months after the second allo-HSCT. The two patients (#7 and #13) who developed graft failure were rescued following a second allo-HSCT from a MUD and were also alive and in remission nine and 2 months later, respectively. Only patient #11, who developed BO after treatment, died of pulmonary infection (Table 1). Overall, the estimated 2-year OS and EFS after allo-HSCT were 90.9 ± 8.7% and 67.0 ± 13.7%, respectively (Figure 3).

## Discussion

Various studies have demonstrated that early MC in patients undergoing allo-HSCT with MAC may be suggestive of a relapse. The relapse rate can be as high as 70–90% for patients with reduced donor chimerism, whereas it is only 10–35% for patients with FDC (8–11). In patients with AML/MDS, testing for mixed T lymphocyte chimerism at day +90 to 120 after allo-HSCT is reported to be a promising approach for detecting patients with pending relapse, while preemptive DLI to maintain full donor T cell chimerism may prevent relapse (12). In the setting of acute lymphoblastic leukemia (ALL) after allo-HSCT, based on the comparison of lineage-sorted donor cell chimerism and quantitative PCR analysis of disease-specific genetic rearrangements to detect MRD, Wethmar et al. demonstrated that two measurements were similarly and complementarily effective as indicators of hematological relapse (50 vs. 4%, respectively; *p* < 0.0001) and decreased OS (47 vs. 87%, respectively; *p* = 0.004) (26). Terwey et al. also showed that, besides its role in MRD monitoring, MC analysis could additionally provide information for impending relapse. Integrating MRD and chimerism analysis allowed for optimal clinical judgment and decision-making to reduce relapse rates through preemptive interventions (27).

In this study, we focused on a subgroup of patients exhibiting MC of CD3+ T cells either in bone marrow or in peripheral blood, and who were MRD-negative, based on flow cytometry and/or molecular analysis early after allo-HSCT with MAC. In patients presenting with MC, several clinical outcomes can be expected. For instance, some may spontaneously recover to FDC without intervention or remain with stable MC without relapse. An increased loss of donor chimerism with the development of neutropenia or pancytopenia, leading to graft failure, may also occur, while most patients with persistent or increased levels of MC may eventually become MRD-positive and experience disease relapse. To prevent MC-associated relapse and/or graft failure, immune modulation such as RWIS and/or DLI are routinely considered. Based on multivariate analysis, a recent report demonstrated that two consecutive increases in MC in the peripheral blood of patients was a strong indicator for relapse (*p* < 0.0001) and immunomodulatory strategies such as RWIS or DLI could significantly decrease this relapse rate (15.7 vs. 57.6%, *p* = 0.0007) (28). Notably, immune modulation is limited in patients with previous grade III–IV aGvHD or with moderate to severe cGvHD. The rate of new-onset GvHD can be as high as ~40%, including grade II–IV aGVHD- or moderate to severe cGVHD. With DLI, GvHD-associated mortality ranges from 4 to 7% (29, 30).

In recent years, HMAs have been shown to exert significant immunomodulatory effects, and clinical studies have been undertaken to assess the usefulness of HMAs for the treatment or prevention of relapse in patients with AML or MDS after allo-HSCT (14, 18–20). In this pilot study, we focused on such a group of patients, who were considered to have the potential for an increased risk of relapse or graft loss, but not necessarily imminent relapse. To rule out the possible effects of other immunomodulatory therapies, RWIS and/or DLI were not implemented in the enrolled patients.

Of the 14 enrolled patients, 11 achieved a FR (9 achieved a CR and 2 a MR) after one to three cycles of LD-DAC treatment. Despite a speedy MR after initial treatment, one patient rapidly experienced secondary graft failure following preemptive therapy for CMV reactivation, while another patient displayed a PR, with skin rash as a cGvHD. Only one patient, who presented with a very low level of donor chimerism (<60%), failed to respond to LD-DAC treatment and rapidly developed secondary graft failure. In terms of safety, the treatment was well tolerated with exerted limited hematological and nonhematological toxicities. More importantly, only two patients developed new symptoms of cGVHD several days or months after the treatment. Most of the patients who achieved and maintained FDC remained alive and in remission without significant clinical complications. Adverse events, including treatment failure, severe GvHD, relapse, and NRM, were documented in only four patients and were acceptable based on continuous monitoring for toxicity using Pocock-type boundary. These data suggested that LD-DAC monotherapy has potential as a treatment option for patients with MC and who are MRD-negative.

There were still questions unanswered regarding to the LD-DAC treatment particularly the influence of diseases (myeloid vs. lymphoid malignancies), donor type (MSD vs. MUD or haplo), GVHD prophylaxis protocol (PTCY vs. CNI-based regimen or ATG vs. no ATG), GVHD status (previous GVHD vs. No GVHD), and the immunosuppressive treatment at LD-DAC treatment (No IS vs. ongoing IS).

The small number of patients enrolled limited the ability to draw definitive conclusions. However, ~80% of the patients responded favorably to LD-DAC treatment with acceptable toxicity, suggesting that LD-DAC may be a potential alternative to RWIS and DLI. Additional prospective studies with larger sample sizes are warranted to confirm the clinical benefits of LD-DAC.

## Data Availability Statement

The original contributions presented in the study are included in the article/Supplementary Material, further inquiries can be directed to the corresponding author/s.

## Ethics Statement

The studies involving human participants were reviewed and approved by Ruijin Hospital Ethics Committee, Shanghai Jiao Tong University School of Medicine. Written informed consent to participate in this study was provided by the participants' legal guardian/next of kin.

## Author Contributions

LW, L-nW, JH, and J-lJ conceived and designed the study and acquired, analyzed, and interpreted the data. LW, L-nW, and J-fZ carried out statistical analysis. LW and L-nW prepared the manuscript. LW, L-nW, J-fZ, W-hG, C-hJ, WT, W-lZ, JH, and J-lJ edited and reviewed the manuscript. All authors contributed to the article and approved the submitted version.

## Conflict of Interest

The authors declare that the research was conducted in the absence of any commercial or financial relationships that could be construed as a potential conflict of interest. The handling Editor declared a past co-authorship with one of the authors JH.

## References

[1] Wang Y Chen H Chen J Han M Hu J Jiong Hu. The consensus on the monitoring, treatment, and prevention of leukemia relapse after hematopoietic stem cell transplantation in China. Cancer Lett. (2018) 438:63–75. 10.1016/j.canlet.2018.08.030 30217562

[2] Kröger N Bacher U Bader P Böttcher S Borowitz MJ Dreger P. NCI first international workshop on the biology, prevention and treatment of relapse after allogeneic hematopoietic stem cell transplantation: report from the committee on disease-specific methods and strategies for monitoring relapse following allogeneic stem cell transplantation. Part I: methods, acute leukemias and myelodysplastic syndromes. Biol Blood Marrow Transplant. (2010) 16:1187–211. 10.1016/j.bbmt.2010.06.008 20558311 PMC7272718

[3] Tsirigotis P Byrne M Schmid C Baron F Ciceri F Esteve J. Relapse of AML after hematopoietic stem cell transplantation: methods of monitoring and preventive strategies. A review from the ALWP of the EBMT. Bone Marrow Transplant. (2016) 51:1431–8. 10.1038/bmt.2016.167 27295272

[4] Thiede C Bornhäuser M Oelschlägel U Brendel C Leo R Daxberger H. Sequential monitoring of chimerism and detection of minimal residual disease after allogeneic blood stem cell transplantation (BSCT) using multiplex PCR amplification of short tandem repeat-markers. Leukemia. (2001) 15:293–302. 10.1038/sj.leu.2401953 11236950

[5] Alizadeh M Bernard M Danic B Dauriac C Birebent B Lapart C. Quantitative assessment of hematopoietic chimerism after bone marrow transplantation by real-time quantitative polymerase chain reaction. Blood. (2002) 99:4618–25. 10.1182/blood.V99.12.4618 12036896

[6] Hoffmann JC Stabla K Burchert A Volkmann T Bornhäuser M Thiede C. Monitoring of acute myeloid leukemia patients after allogeneic stem cell transplantation employing semi-automated CD34+ donor cell chimerism analysis. Ann Hematol. (2014) 93:279–85. 10.1007/s00277-013-1961-4 24352219

[7] Lamba R Abella E Kukuruga D Klein J Savasan S Abidi MH. Mixed hematopoietic chimerism at day 90 following allogenic myeloablative stem cell transplantation is a predictor of relapse and survival. Leukemia. (2004) 18:1681–86. 10.1038/sj.leu.2403468 15318247

[8] Barrios M Jiménez-Velasco A Román-Gómez J Madrigal ME Castillejo JA Torres A. Chimerism status is a useful predictor of relapse after allogeneic stem cell transplantation for acute leukemia. Haematologica. (2003) 88:801–10. 12857560

[9] Huisman C de Weger RA de Vries L Tilanus MG Verdonck LF. Chimerism analysis within 6 months of allogeneic stem cell transplantation predicts relapse in acute myeloid leukemia. Bone Marrow Transplant. (2007) 39:285–91. 10.1038/sj.bmt.1705582 17262061

[10] Reshef R Hexner EO Loren AW Frey NV Stadtmauer EA Luger SM. Early donor chimerism levels predict relapse and survival after allogeneic stem-cell transplantation with reduced intensity conditioning. Biol Blood Marrow Transplant. (2014) 20:1758–66. 10.1016/j.bbmt.2014.07.003 25016197 PMC4194246

[11] Horn B Petrovic A Wahlstrom J Dvorak CC Kong D Hwang J. Chimerism-based pre-emptive immunotherapy with fast withdrawal of immunosuppression and donor lymphocyte infusions after allogeneic stem cell transplantation for pediatric hematologic malignancies. Biol Blood Marrow Transplant. (2015) 21:729–37. 10.1016/j.bbmt.2014.12.029 25644958

[12] Lee HC Saliba RM Rondon G Chen J Charafeddine Y Medeiros LJ. Mixed T Lymphocyte chimerism after allogeneic hematopoietic transplantation is predictive for relapse of acute myeloid leukemia and myelodysplastic syndromes. Biol Blood Marrow Transplant. (2015) 21:1948–54. 10.1016/j.bbmt.2015.07.005 26183077 PMC4604040

[13] Zeidan AM Forde PM Symons H Chen A Smith BD Pratz K. HLA-haploidentical donor lymphocyte infusions for patients with relapsed hematologic malignancies after related HLA-haploidentical bone marrow transplantation. Biol Blood Marrow Transplant. (2014) 20:314–8. 10.1016/j.bbmt.2013.11.020 24296490 PMC4010132

[14] Luznik L Fuchs EJ. Donor lymphocyte infusions to treat hematologic malignancies in relapse after allogeneic blood or marrow transplantation. Cancer Control. (2002) 9:123–37. 10.1177/107327480200900205 11965233

[15] Leko V Varnum-Finney B Li HZ Gu YS Flowers D Nourigat C. SIRT1 is dispensable for function of hematopoietic stem cells in adult mice. Blood. (2012) 119:1856–60. 10.1182/blood-2011-09-377077 22219225 PMC3293640

[16] Sugiyama A Yujiri T Tanaka M Tanaka Y Nakamura Y Tanizawa Y. SIRT1 is downregulated during peripheral blood stem cell mobilization in healthy donors by granulocyte-colony stimulating factor. Ann Hematol. (2016) 95:1381–2. 10.1007/s00277-016-2701-3 27220637

[17] Long J Chang L Shen Y Gao WH Wu YN Dou HB. Valproic acid ameliorates graft-versus-host disease by downregulating Th1 and Th17 Cells. J Immunol. (2015) 195:1849–57. 10.4049/jimmunol.1500578 26179902

[18] Schroeder T Rautenberg C Haas R Germing U Kobbe G. Hypomethylating agents for treatment and prevention of relapse after allogeneic blood stem cell transplantation. Int J Hematol. (2018) 107:138–50. 10.1007/s12185-017-2364-4 29143282

[19] Choi J Ritchey J Prior JL Holt M Shannon WD Deych E. In vivo administration of hypomethylating agents mitigate graft-versus-host disease without sacrificing graft-versus-leukemia. Blood. (2010) 116:129–39. 10.1182/blood-2009-12-257253 20424188 PMC2904576

[20] Lindblad KE Goswami M Hourigan CS Oetjen KA. Immunological effects of hypomethylating agents. Expert Rev Hematol. (2017) 10:745–52. 10.1080/17474086.2017.1346470 28644756 PMC6071309

[21] Gao L Zhang YQ Wang SB Kong PY Su Y Hu J. Effect of rhG-CSF combined with decitabine prophylaxis on relapse of patients with high-risk MRD-negative AML after HSCT: an open-label, multicenter, randomized controlled trial. J Clin Oncol. (2020) 38:4249–59. 10.1200/JCO.19.03277 33108244 PMC7768335

[22] Nollet F Billiet J Selleslag D Criel A. Standardisation of multiplex fluorescent short tandem repeat analysis for chimerism testing. Bone Marrow Transplant. (2001) 28:511–8. 10.1038/sj.bmt.1703162 11593326

[23] Jung SH Lee TY Kim KM George SL. Admissible two-stage designs for phase II cancer clinical trials. Stat Med. (2004) 23:561–9. 10.1002/sim.1600 14755389

[24] Ivanova A Qaqish BF Schell MJ. Continuous toxicity monitoring in phase II trials in oncology. Biometrics. (2005) 61:540–5. 10.1111/j.1541-0420.2005.00311.x 16011702

[25] Kaplan EL Meier P. Nonparametric estimation from incomplete observations. J Am Stat Assoc. (1958) 53:457–80. 10.1080/01621459.1958.10501452

[26] Wethmar K Matern S Eßeling E Angenendt L Pfeifer H Brüggemann M. Monitoring minimal residual/relapsing disease after allogeneic haematopoietic stem cell transplantation in adult patients with acute lymphoblastic leukaemia. Bone Marrow Transplant. (2020) 55:1410–20. 10.1038/s41409-020-0801-0 32001801

[27] Terwey TH Hemmati PG Nagy M Pfeifer H Gökbuget N Brüggemann M. Comparison of chimerism and minimal residual disease monitoring for relapse prediction after allogeneic stem cell transplantation for adult acute lymphoblastic leukemia. Biol Blood Marrow Transplant. (2014) 20:1522–9. 10.1016/j.bbmt.2014.05.026 24907626

[28] Jacque N Nguyen S Golmard JL Uzunov M Garnier A Leblond V. Chimerism analysis in peripheral blood using indel quantitative real-time PCR is a useful tool to predict post-transplant relapse in acute leukemia. Bone Marrow Transplant. (2015) 50:259–65. 10.1038/bmt.2014.254 25387089

[29] Caldemeyer LE Akard LP Edwards JR Tandra A Wagenknecht DR Dugan MJ. Donor lymphocyte infusions used to treat mixed-chimeric and high-risk patient populations in the relapsed and nonrelapsed settings after allogeneic transplantation for hematologic malignancies are associated with high five-year survival if persistent full donor chimerism is obtained or maintained. Biol Blood Marrow Transplant. (2017) 23:1989–97. 10.1016/j.bbmt.2017.07.007 28712934

[30] Bar M Flowers MED Storer BE Chauncey TR Pulsipher MA Thakar MS. Reversal of low donor chimerism following hematopoietic cell transplantation using pentostatin and donor lymphocyte infusion: a prospective phase II multicenter trial. Biol Blood Marrow Transplant. (2018) 24:308–13. 10.1016/j.bbmt.2017.10.016 29032276 PMC5767527

