# Supplementary material for: Low-Dose Decitabine Monotherapy Reverses Mixed Chimerism in Adult Patients After Allogeneic Hematopoietic Stem Cell Transplantation With Myeloablative Conditioning Regimen: A Pilot Phase II Study
Source: Front Med (Lausanne). 2021 Feb 23;8:627946. doi: 10.3389/fmed.2021.627946 (PMC7940531; doi:10.3389/fmed.2021.627946)
Supplement: Supplementary file 1 [file Table_1.DOCX]

Supplementary Material

**Table S1. Simon’s stage II design of sample size***

| **n** | **n_1_** | **r_1_** | **r_2_** | **Type 1 Error** | **Power** | **EN_0_** | **Probability of early stopping** | **Interval for w** | **Comment** |
| --- | --- | --- | --- | --- | --- | --- | --- | --- | --- |
| 12 | 10 | 7 | 8 | 0.0437 | 0.8140 | 10.1 | 0.9453 | 0.7673, 1 | Minimax |
| 13 | 4 | 2 | 9 | 0.0383 | 0.8209 | 6.8 | 0.6875 | 0, 0.7672 | Optimal |

** n* as the total number of patients should be enrolled; *n1*_1_ as the number of patients accrued during stage I; *r*_1_, if *r*_1_ or fewer patients with favorable response are observed during stage I, the trial is stopped early for futility; *r*_2_, if *r*_2_ or fewer patients with favorable response are observed by the end of stage II, then no further clinical trial is warranted. *EN*_0_ is the expected sample size for the trial when response rate is *p*_0_ Interval for *w* is the set of values *w* such that the design minimizes *w * n + (1 – w) * EN*_0_

**Table S2. Continuous monitoring for toxicity using Pocock-type boundary***

| **No of Patients, n** | 1 | 2 | 3 | 4 | 5 | 6 | 7 | 8 | 9 | 10 | 11 | 12 | 13 |
| --- | --- | --- | --- | --- | --- | --- | --- | --- | --- | --- | --- | --- | --- |
| **Boundary, bn** | - | 2 | 2 | 3 | 3 | 3 | 3 | 4 | 4 | 4 | 4 | 5 | 5 |

* Sequential boundaries will be used to monitor severe event. The accrual will be halted if the number of events is equal to or exceeds bn out of n patients with full follow-up.
